# Storage of Correlated Patterns in Standard and Bistable Purkinje Cell Models

**DOI:** 10.1371/journal.pcbi.1002448

**Published:** 2012-04-26

**Authors:** Claudia Clopath, Jean-Pierre Nadal, Nicolas Brunel

**Affiliations:** 1 Laboratory of Neurophysics and Physiology, CNRS and Université Paris Descartes, Paris, France; 2 Laboratoire de Physique Statistique (CNRS, ENS, UPMC, Univ. Paris Diderot), Ecole Normale Supérieure, Paris, France; 3 Centre d'Analyse et de Mathématique Sociales (CNRS, EHESS), Ecole des Hautes Etudes en Sciences Sociales, Paris, France; Indiana University, United States of America

## Abstract

The cerebellum has long been considered to undergo supervised learning, with climbing fibers acting as a ‘teaching’ or ‘error’ signal. Purkinje cells (PCs), the sole output of the cerebellar cortex, have been considered as analogs of perceptrons storing input/output associations. In support of this hypothesis, a recent study found that the distribution of synaptic weights of a perceptron at maximal capacity is in striking agreement with experimental data in adult rats. However, the calculation was performed using random uncorrelated inputs and outputs. This is a clearly unrealistic assumption since sensory inputs and motor outputs carry a substantial degree of temporal correlations. In this paper, we consider a binary output neuron with a large number of inputs, which is required to store associations between temporally correlated sequences of binary inputs and outputs, modelled as Markov chains. Storage capacity is found to increase with both input and output correlations, and diverges in the limit where both go to unity. We also investigate the capacity of a bistable output unit, since PCs have been shown to be bistable in some experimental conditions. Bistability is shown to enhance storage capacity whenever the output correlation is stronger than the input correlation. Distribution of synaptic weights at maximal capacity is shown to be independent on correlations, and is also unaffected by the presence of bistability.

## Author Summary

The cerebellum is one of the main brain structures involved in motor learning. Classical theories of cerebellar function assign a crucial role to Purkinje cells (PCs), that are assumed to perform as simple perceptrons. In these theories, PCs should learn to provide an appropriate motor output, given a particular input, encoded by the granule cell (GC) network. This learning is assumed to occur through modifications of  synapses, under the control of the climbing fiber input to PCs, which is supposed to carry an error signal. In this paper, we compute storage capacity and distribution of weights in the presence of temporal correlations in inputs and outputs, which are unavoidable in sensory inputs and motor outputs. Furthermore, we study how bistability in the PCs affects capacity and distribution of weights. We find that (1) capacity increases monotonically with both input and output correlations; (2) bistability increases storage capacity, when the output correlation is larger than the input correlation; (3) the distribution of weights at maximal capacity is independent of the degree of temporal correlations, as well as the nature of the output unit (mono or bistable) and is in striking agreement with experimental data.

## Introduction

**Figure 1:**
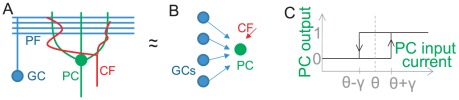
Simplified model of Purkinje cell. A. Simplified sketch of the cerebellar cortex circuit. GC stands for Granule cell, PC for Purkinje cell, PF for Parallel fiber, CF for Climbing fiber. B. Perceptron model: the input layer is composed of GCs, the output unit is the PC. CF represents the error signal. C. Bistable output. If the previous output is 0, the input current needs to be larger than 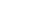 to switch the output to 1. If the previous output is 1, the input current needs to be below 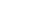 to switch the output to 0.

The cerebellum is heavily involved in learning tasks that requires precise spatio-temporal sequences, such as grasping, precise eye movement, etc. It has long been thought [1], [2] that the particular form of learning at work in this structure is supervised learning, whereby the neural system adapts its synaptic weights to reproduce a desired input-output relationship, thanks to an error signal. As such, the cerebellum would be one of the main structures of the central nervous system involved in supervised learning [3]. More precisely, it has been proposed [1], [2] that each Purkinje cell (PC) may be seen as a single layer perceptron [4], [5] - a single binary output neuron, with its 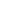 input synapses (see Figure 1). Indeed, the PCs, the sole output of the cerebellar cortex, receive two types of excitatory synaptic inputs: individually weak synaptic inputs from a large number (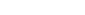) of Granule cells (GCs), through the Parallel Fibers (PFs); and a single, very strong input from the inferior olive, through the so-called Climbing Fiber (CF). This strong input is thought to represent the ‘error signal’ similarly to a perceptron - indeed, CF firing rates are in some conditions modulated by the error made by an animal [6], and it has been shown in vitro that CF activity affects synaptic plasticity [7], [8].

On the theoretical side, a particularly well studied problem is the one of learning random input-output associations by the perceptron. The maximal storage capacity (maximal number of random associations that can be learned per input synapse, in the large 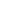 limit) has been computed by several methods [9], [10], [11]. For binary input-output units, unconstrained synaptic weight, and random unbiased associations, the maximal capacity is 2, i.e. the number of associations that can be stored is two times the number of inputs. If synaptic weights are sign-constrained, as one expects in real neurons, the capacity is divided by a factor 2 and becomes equal to 1 [12], [13], [14]. The capacity has also been computed in the presence of robustness constraints, biased associations, and other constraints on synaptic weights [10], [15]. Distributions of synaptic weights at the maximal capacity can also be computed. At maximal capacity, the distribution is a Gaussian when weights are unconstrained, while sign constraints lead to truncated Gaussian distributions, together with a delta function at zero weight synapses [16], [17]. Brunel et al. [17] showed that the distribution of 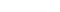 synaptic weights is in very good agreement with the analytically computed distribution for a perceptron close to maximal capacity, giving further support to the idea that PCs are similar to perceptrons.

The study of Brunel et al. [17] considered for simplicity uncorrelated input-output associations. In the case of the cerebellum, the assumption of uncorrelated inputs and outputs is clearly unrealistic, as any naturalistic sensory input or sequence of motor commands will carry a substantial degree of temporal correlations. Moreover, under some conditions, PC dynamics seem to be consistent with a bistable device [18], [19], [20], [21], [22], [23]. The consequences of temporal correlations, as well as the presence of bistability on the learning capacity of the model remain however to be clarified.

In this paper, we study the capacity and optimal connectivity in a perceptron network storing correlated input-output associations. More precisely, we study (i) a standard binary perceptron, whose task is to learn a sequence of associations with an arbitrary level of temporal correlations in the inputs and outputs; (ii) a bistable perceptron, again subjected to a correlated sequence of associations. We show that the capacity (maximal number of associations in a learnable sequence) is independent of the correlations in the output if the inputs are not correlated. If the inputs are temporally correlated, the capacity grows with output correlation. The capacity diverges in the limit when both correlations become close to unity. The weight distribution is shown to be independent of the degree of correlation, both in the input and output. It is also found that adding a bistability range increases capacity when the output correlation is larger than the input correlation. The optimal width of the bistability range increases with output correlation. Finally, we show that in order to reach maximal capacity, the error signal (CF) has to change the state of the output unit (PC) in addition to its synapses, consistent with experimental data [20], [18].

## Results

### Binary perceptron with correlations

In this section, we investigate storage of associations between temporally correlated input and output sequences. The maximal capacity is defined as the maximal length of a sequence that can be learned per input synapse, or in other words the maximal number of associations composing the sequence. We study a simple Markov chain model for generating the sequences. The sequence to be learned is composed of 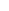 patterns, 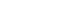. A pattern is given by the state of input cell 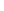 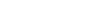, 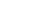 (Granule cell) and the state of the target output sequence, 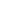 (Purkinje cell, 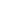 for target). The patterns are presented always in the same order. For the first pattern in the sequence, 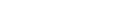, 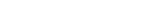, where 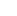 is the input coding level, i.e. the probability that the granule cell is active in a given pattern. For the following patterns, we have
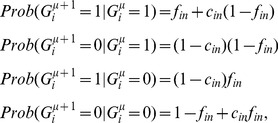
(1)where 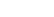 measures the correlation between successive input patterns. Note that different input neurons are not correlated. The target outputs 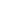 are generated similarly but with probability 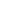 and correlation 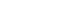. In most of the paper we chose 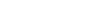, unless stated otherwise.

In the perceptron, the output is obtained though a comparison of a weighted sum of the inputs to a threshold,
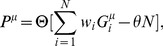
(2)where 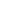 are the synaptic weights and 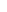 is the threshold. The Heaviside function 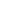 is 1 if the argument positive and zero otherwise.

Correlations defined by Equation 1 make calculations using the replica method [10], [15] extremely involved. The only case in which calculations can be performed easily is with 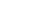. In this case, one can show that both capacity and distribution of weights are independent of the output correlation. In the more general case, 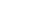, we resort to numerical simulation.

For numerical simulations, we chose the variant of the perceptron algorithm used in Brunel et al. [17]. Namely, the threshold being fixed, the weights are modified according to the standard perceptron learning rule, i.e.
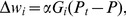
(3)where 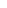 is the learning rate, except that the weights have a lower hard bound at 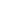.

This rule can be shown to be guaranteed to converge to a solution, provided the solution exists, and 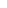 is small enough (see Methods). Interestingly, the rule is in agreement with basic experimental protocols leading to plasticity in slice experiments [8]. Indeed, LTD is induced when the CF and the PF are simultaneously active (CF firing more than its average firing rate 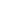) and LTP when PF fires alone (meaning that CF does not fire, i.e. below 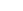). The plasticity can be written as 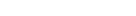. It was used to model cerebellar learning in tasks such as the Vestibulo-Ocular Reflex (VOR) adaptation [24], [25], [26]. This learning rule can easily be mapped to the perceptron learning rule as the CF is thought to signal the error 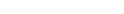 [1], [2].

**Figure 2:**
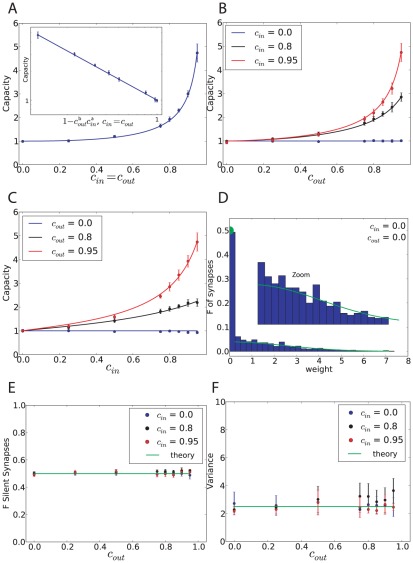
Standard perceptron storing correlated input/output sequences. A. Maximal capacity as a function of 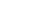 (dot: simulation, error bar: standard deviation, line: fit with a function 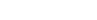). Inset. Same but plotted as a function of 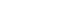 in a loglog scale. B. Maximal capacity as a function of the output correlation for different input correlations. C. Maximal capacity as a function of the input correlation for different output correlations. D. Weight distribution after learning at the maximal capacity for the case of uncorrelated input and output (blue: simulation, green: theory). The theoretical fraction of silent synapses is 0.5. The rest of the distribution is a truncated Gaussian with zero mean and standard deviation 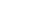. E. Fraction of silent synapses as a function of the output correlation for different input correlations. The theoretical value is 0.5 (green). F. Variance of the weight distribution normalized by the mean synaptic weight, fitted by a truncated Gaussian. The theoretical value is 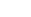 (green). In all simulations, the perceptron has 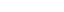 inputs and the simulations were averaged over 10 trials. The coding level is 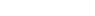.

Figure 2 shows the capacity and distribution of synaptic weights of a binary perceptron storing associations of correlated input/output sequences, for 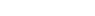. If the inputs are uncorrelated, the maximal capacity is independent of the output correlation and is equal to 1, as shown analytically (Figure 2B, blue line). This can be understood easily since the classification problem would not change after reshuffling the pattern index 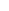. Second, we find numerically that the capacity is also constant and equal to 1 for uncorrelated inputs and correlated outputs (Figure 2C, blue line). This means that if the output is temporally uncorrelated, temporal correlation in the input does not affect the number of associations the system can learn. However, if the inputs are correlated, the capacity increases with output correlation. We find that the capacity can be well fitted by the function
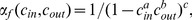
(4)with 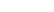, 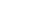, 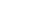. The intuitive reason is that if the patterns are highly correlated, they become more similar to one another, and thus it is easier to learn them.

Simulations (Figure 2E–F) indicate that the weight distribution at maximal capacity is a truncated Gaussian with 50% of silent synapses, independent of the level of both input and output correlations. This finite fraction of silent synapses is due to the constraint that synapses cannot become negative. During the learning process, some synapses tend to go up, others tend to go down. Some would tend to go to negative values, but become stuck at zero. As one reaches the maximal capacity, a finite fraction of these synapses ends up exactly at zero, while the remaining synapses are distributed according to a truncated Gaussian [17].

**Figure 3:**
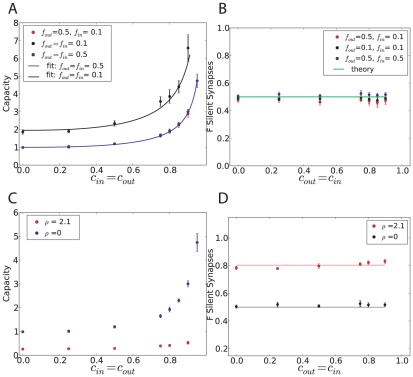
Standard perceptron storing correlated input/output sequences with various input and output coding levels as well as robustness parameters. A–B. Dependence on coding levels. A. Maximal capacity as a function of 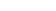 for different coding levels (symbols: simulations, error bar: standard deviation). The blue curve shows the fit used for Figure 2, Equation 4. The black curve shows the blue line multiplied by the capacity for uncorrelated patterns with 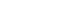. No robustness constraint is considered. B. Fraction of silent synapses as a function of 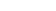 for different coding levels, no robustness constraint. The theoretical value is 0.5 (green). C–D. Dependence on the robustness parameter. C. Capacity as a function of same input and output correlation for different robustness parameters (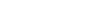). D. Fraction of silent synapses as a function of same input and output correlation for different robustness parameters (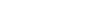). In all simulations, the perceptron has 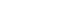 inputs. Means and standard deviations were computed from 10 independent samples.

We have so far focused on the case 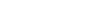. This is at odds with available data on the activity of granule cells and Purkinje cells in vivo, that shows consistently high firing rates in Purkinje cells, while granule cells tend to fire at much lower rates [27]. In Figure 3 therefore, we show how the capacity and the number of silent synapses depend on the input and output coding levels. We find that the capacity is independent on the input coding level, but strongly depends on the output coding level, for any correlation level. The capacity increases if the output coding level decreases, and diverges in the limit of a sparse output coding level [10]. For example, when 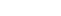, the capacity is approximately doubled compared to the case 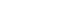. Interestingly, the capacity is well fitted by a function which is a product between two terms, one which depends only on 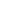, the other only on correlations, 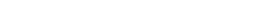 where 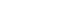 is given by Equation 4. The number of silent synapses is found to be independent on input and output coding levels (Figure 3B), and is therefore independent on all statistical parameters characterizing the associations.

Experimentally, the fraction of silent synapses was estimated to be about 80% [28]. The fraction of silent synapses is 50% when no robustness constraints are imposed on learning, but it increases if a robustness constraint is introduced [17]. The robustness parameter 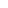 is defined in the following way: for a robust classification, we now need 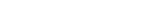 if 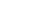 and 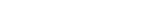 if 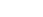. In Figure 3 C–D, we show, consistent with previous studies with uncorrelated patterns [10], [17], that the capacity decreases when the robustness constraint increases, whereas the fraction of silent synapses increases. Note that for 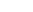, the capacity can no longer be expressed as a simple product of the capacity for uncorrelated patterns, times 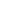. The increase in capacity as the input and output correlations increase is relatively less pronounced than for 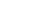. For 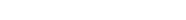, 80% of silent synapses are found [17], consistent with the experimental estimate [28]. This fraction is again independent on both input and output correlation, as shown in Figure 3D.

### Bistable perceptron

#### Bistable perceptron with correlations in the output and uncorrelated inputs

In *in vivo* experiments, PCs undergo under some conditions transitions between so-called up and down states. These up and down states are thought to be a manifestation of an intrinsic bistability of the PCs [18], [20], [22], [23] but see [29]. The computational advantage of bistability in PCs remains however an open question. We argue here that bistable PCs can serve to increase memory storage if the correlation in the output is larger than the correlation in the input. More precisely, we use a binary perceptron where the output is bistable, i.e. it depends on past history: to switch the cell from 0 to 1, the input current should be larger than 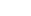, while to switch it from 1 to 0, it should be smaller than 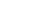. Hence, 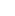 is the size of the bistable range (see Figure 1B). For the patterns to be learned, we now need to find synaptic weights 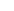 such that
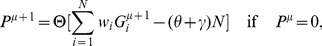
(5)

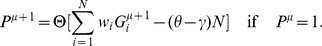
(6)


To investigate how the capacity depends on temporal correlations in the output, we consider sequences of patterns generated from a Markov chain as defined in the previous section, Equation 1.

**Figure 4:**
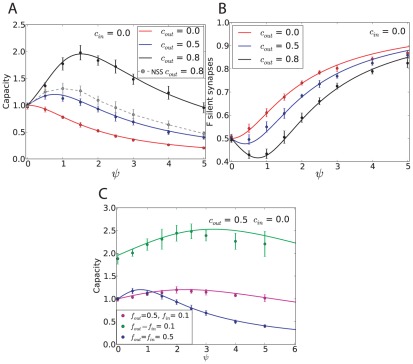
Bistable perceptron with correlated output, but no input correlation. A. Maximal capacity as a function of the bistability range 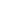, for different values of the output correlation 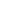 with 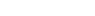 (line: theoretical results, symbols: simulations, error bars: standard deviation). The grey line shows the results of simulations with a different learning rule where the CF does not change the state of the PC, called the “no state switching” rule (NSS)(see Methods section). B. Fraction of silent synapses as a function of 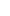 for different values of 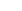 with 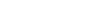. C. Maximal capacity as a function of the bistability range 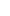, for different values of coding level and 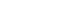. 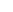 is defined as 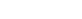 (see Methods). For the simulations, the network is composed of 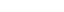 inputs. Means and standard deviations were computed from 10 independent samples.

The analytical calculation for correlated output and uncorrelated inputs (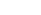) is described in the Method section in detail. Both capacity and distribution of synaptic weight are computed using the replica method [10], [15], [16], [17]. The results are shown in Figure 4. For a given value of output correlation 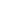, there is an optimal bistable range that maximizes the capacity. When correlations are present in the output, the probability that the state of the cell remains unchanged from one pattern to the next is higher than the probability that it changes. Bistability tends to favor stability of the output in its previous state, and thus makes it easier for the system to learn such input/output associations.

Figure 4 also shows that the maximal capacity at the optimal bistable range grows with output correlation. Furthermore, the optimal bistable range also grows with output correlations - so that if the target outputs are highly correlated, the best strategy is to have a large bistabile range. Conversely, the optimal 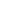 is equal to zero for 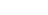. The weight distribution has the same stereotypical form as in the standard case with a large number of silent synapses. Interestingly, for any output correlation, the fraction of silent synapses is constant and equal to 50% at the optimal bistable range (see Figure 4B). Here no robustness constraint is considered.

Figure 4C shows how the capacity depends on input and output coding levels. As expected, the capacity is increased when the output coding level decreases. Interestingly, for a fixed bistable range, the capacity also depends on the input coding level. The optimal bistable range increases when the input coding level decreases. However, the capacity at the optimal bistable range is independent on the input coding level.

We then numerically confirm the theoretical results using a perceptron learning algorithm (Figure 4). The learning rule is defined as previously (Equation 3, with the same constraints on the weights and threshold). However, here the error signal not only influences the weight change but also the state of the output. The output therefore switches to match the target output if there is an error after each pattern presented. Then, when the next pattern is presented, the output depends on the previous pattern which is guaranteed to be correct (see Method section for details).

If the CF does not change the state of the PCs, the simulations does not reach maximal capacity (Figure 4A, grey dashed line). The intuitive reason is that, if the current PC state is wrong, the next state is going to be wrongly influenced by the wrong current state due to bistability.

#### Bistable perceptron with input/output correlations

**Figure 5:**
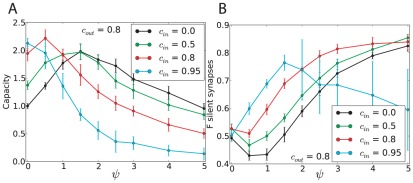
Bistable perceptron with correlated input/output. A. Capacity as a function of 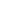 for 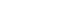 and different 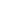. B. Fraction of silent synapses at the maximal capacity as a function of 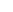 for 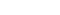 and for different 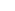. 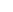 is defined as 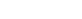. The network is composed of 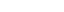 inputs with 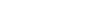. Simulations were repeated 10 times (error bar: standard deviation).

In this section, we simulate numerically the bistable perceptron with correlated input and output (Figure 5). When correlation in the input increases, the optimal bistable range decreases. Intuitively, temporal correlations in the input will automatically produce temporal correlations in the output. Therefore, if the correlation in the input is stronger, a smaller bistability is needed. Additionally, when correlation in the input is higher than the correlation in the output, the maximal capacity is maximized without bistability. Capacity is therefore enhanced through bistability only if the correlation in the output is larger than the correlation in the input. Again, this is understood by the fact that bistability introduces naturally more correlations in the output than what is in the input.

## Discussion

In this paper, we reconsidered the problem of learning random input-output associations in a perceptron with excitatory weights, considered as a model for cerebellar Purkinje cells. We computed the storage capacity, and distribution of synaptic weights, in two distinct models that are subjected to correlated input-output associations, described as Markov chains: a standard binary perceptron; and a bistable perceptron.

We find that the maximal capacity increases monotonically when both input and output correlations are increased. The capacity diverges in the limit when both go to unity. This divergence of the capacity is reminiscent of the divergence of the capacity of perceptrons storing uncorrelated input-output associations in the limit when the output coding level 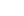 goes to one [10]. In the bistable perceptron, we find that the capacity is optimal for a non-zero bistable range, whenever the output correlation is larger than the input correlation. This result can be understood intuitively by the fact that bistability will automatically generate additional temporal correlations in the output of a neuron. A bistable neuron is therefore better equipped to learn such input/output associations, compared to a standard perceptron.

Interestingly, Purkinje cells are known to exhibit bistability in vitro [22], [20], [19], [23] and their dynamics in vivo has been shown to be compatible with a bistable unit, at least under some conditions [20], [18] (but see [29]). Our results suggest that this bistable behavior might help Purkinje cells to achieve a higher capacity. We further speculate that different areas of the cerebellum might use cells with different degrees of bistability, depending on the temporal correlations imposed upon these areas. Our results also suggests that to optimally use bistability, a learning rule leading to optimal capacity should implement a mechanism that switches the state of the neuron in the case of an error. This switching mechanism fits perfectly with the properties of the climbing fiber (CF) input. Indeed, CF inputs (the putative error signal in PCs) have been able to switch Purkinje cells both from the down to the up state, and from the up to the down state [20], [18].

We also found that the distribution of synaptic weights at the maximal capacity is independent on the degree of correlations in the input and output, for both standard and bistable perceptrons. It is also independent on the input and output coding levels. This distribution is composed of a finite fraction of zero-weight (silent) synapses, and a truncated Gaussian distribution for positive weights. As shown in [17], such a distribution fits very well data from paired recordings in cerebellar slices [28], [17]. Our results suggest that such a distribution might be a universal property of neural systems storing information with excitatory synapses, close to maximal capacity [30].

The learning algorithm that we used is in good qualitative agreement with standard protocols used to induce plasticity in 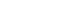 synapses. This algorithm can be proved to converge to a solution of the learning problem, provided such a solution exists (see appendix). For the algorithm to converge, changes induced by an individual pattern must be extremely small (of the order of 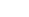 where 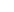 is the number of inputs). It is unclear whether such small changes can be induced at this synapse. If individual synaptic changes are not small, then maximal capacity will not be reached with such an algorithm. It would be interesting to investigate the capacity of algorithms in which synaptic changes are of order 1, rather than of the order of 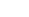.

We have focused on the GC
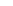
PC feedforward network. Many other sites of plasticity have been identified in the cerebellum, including in the deep cerebellar and medial vestibular nuclei, and in interneurons of the molecular layer that provide feedforward inhibition to PCs (see e.g. [31]). It remains to be investigated how interactions between these different plasticity sites allows the cerebellum to optimize its learning capabilities.

## Methods

### Variant of the perceptron algorithm for positive weights, fixed threshold and 0,1 units: Proof of convergence

The conditions for storing associations can be expressed as,
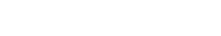


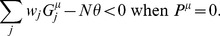
(7)Defining
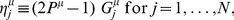
(8)

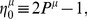
(9)equation (7) can be rewritten as
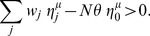
(10)


The constraint on the weights are
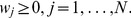
(11)


One can write the perceptron algorithm with sign constraint as:

- (0) 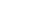; start with 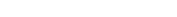;
- (1) pick a pattern 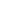 at random; if 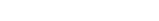 then, for each 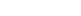,
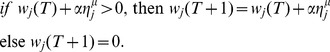
(12)Increase 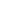 by 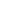 if a change have been made (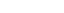). This means that 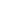 measures the number of presented patterns for which changes had to be made, rather than the total number of presented patterns.
- Go to (1)

The principle of the proof of convergence is as follows. Let us suppose that there exists a solution to the learning task with positive weights. In other words, we assume there exists a set of weights 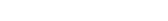 and a stability parameter 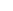 such that for every 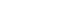


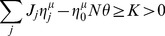
(13)is satisfied.

As in the standard case (with unconstrained weights), one computes the cosine of the angle between the weight vectors 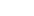 and 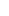:
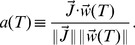
(14)One shows that this quantity increases monotonically with 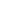, so that it becomes larger than 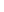, which is not possible: hence after some finite value of 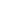 there is no pattern for which a learning step has to be made.

We write 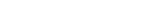 with 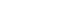 or 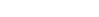 according to (12), 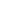 being the pattern learnt at step 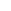,
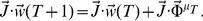
(15)This can be rewritten as
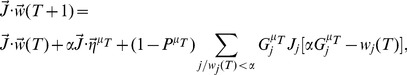
(16)where the last term in the r.h.s. is specific to the learning of patterns for which the desired output is 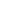.

From the hypothesis that 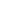 is a solution, one has
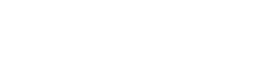
so that
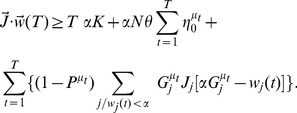
(17)


One proceeds similarly for the norm:
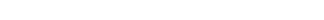


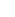
 being either 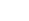 or 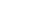 with 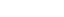 in the later case, one has 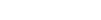, where 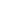 is the maximal fraction of active inputs.

To get a bound on the scalar product 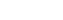 one proceeds as in Equation 16,
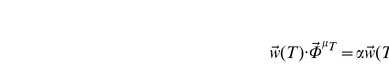
This leads to
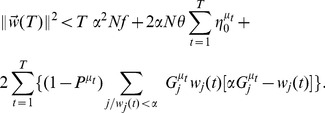
(18)


Since 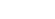, and 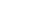 is smaller than 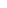 in the sum over 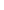,
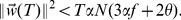
(19)From Equation 18, we have
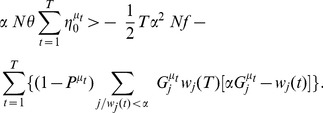
(20)Making use of this inequality, one gets from Equation 17 the bound
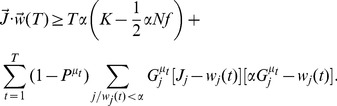
(21)In the sum over 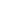, one has 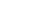 and 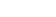, and a contribution only from 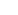 such that 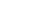. Hence a crude lower bound on this sum is
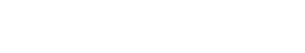
Putting everything together, we find
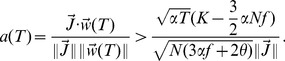
(22)If we choose 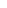 small enough, that is
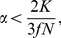
(23)then the right hand side of Equation 22 becomes larger than 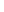 for 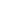 larger than
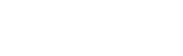
This means that the algorithm converges after a number of learning steps smaller than 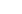.

Note that this proof of convergence of the sign-constrained perceptron is distinct from the one of Amit et al. [12]. Amit et al.consider 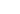 input and output units, and a threshold which is zero. In our case, the units are 0,1, and the threshold is strictly positive. This imposes a constraint on the learning rate 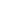, which is not present in the case of Amit et al. [12].

### Calculation of the capacity of a bistable perceptron

The capacity is defined as the maximal number of random associations that can be learned per input synapse. The capacity of a perceptron with bistable output, where the target output is correlated and the inputs are uncorrelated, can be computed analytically, using the replica method [10], [15]. The calculation of weight distribution can also be computed with the same method. Both calculations are similar to the calculations described in the supplementary information of Brunel et al. [17] (called BSI in the following). The idea, introduced by Elizabeth Gardner [10], is to consider the space of all possible couplings. In this space, only a subspace of weights satisfy the constraints imposed by learning. These constraints are
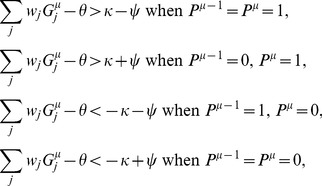
(24)where we have introduce a robustness parameter 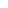 (set to zero in all the results section). The probabilities of the four distinct sets of pairs of successive outputs are
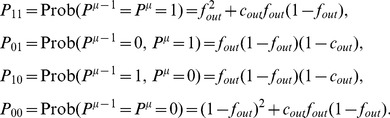
(25)Note that in the large 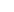 limit, if we take 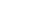, the synaptic weights need to scale as 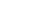, while 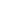 and 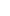 both have to scale as 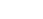. We therefore define 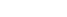 and 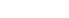.

The ‘typical’ volume of the subspace of weights satisfying Equations 24 can then be computed, as a function of 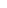. The maximal capacity is obtained as the value of 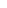 for which the typical volume vanishes. This is done using the replica method. This method consists in calculating the average volume of 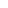 independent replicas of the system (average here means average over the distribution of the stored patterns),
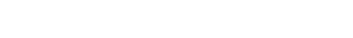
where 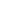 is the stability of pattern 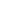 in replica 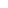, defined as
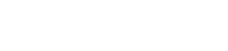
and 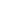 is the Heaviside function that imposes constraints (Equations 24).

The calculation follows a standard procedure. One first introduces integral representations for the Heaviside functions, which allows to average over the patterns. Then, one introduces order parameters
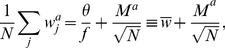
(26)

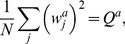
(27)

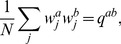
(28)together with conjugate parameters 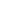, 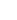 and 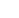. We then use a replica-symmetric ansatz (all the order parameters are taken to be independent of replica index 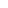), perform the limit 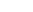 and obtain
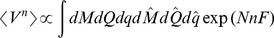
(29)

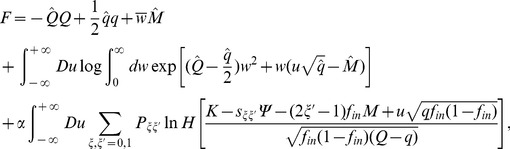
(30)where 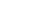 if 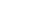, while 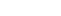 if 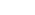, 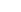 is the Gaussian measure 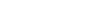 where 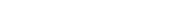, and 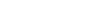.

In the large 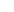 limit, 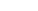, 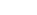. In that limit, we rewrite
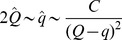
(31)

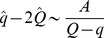
(32)

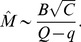
(33)Saddle point equations give in that limit
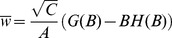
(34)

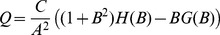
(35)

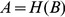
(36)

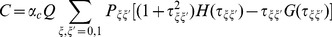
(37)

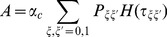
(38)

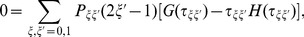
(39)where
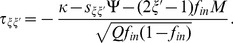
(40)These equations can be solved to numerically to obtain all quantities of interest.

Finally, the equation for the distribution of synaptic weights for the bistable perceptron is identical to the one for the standard perceptron, i.e. at maximal capacity
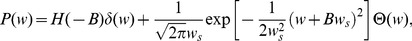
(41)where
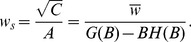
(42)In particular the fraction of zero weight synapses is 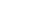.
